# Analysis of Auxin-Encoding Gene Family in *Vigna radiata* and It’s Cross-Species Expression Modulating Waterlogging Tolerance in Wild *Vigna umbellata*

**DOI:** 10.3390/plants12223858

**Published:** 2023-11-15

**Authors:** Chandra Mohan Singh, Shalini Purwar, Akhilesh Kumar Singh, Bhupendra Kumar Singh, Mukul Kumar, Hitesh Kumar, Aditya Pratap, Awdhesh Kumar Mishra, Kwang-Hyun Baek

**Affiliations:** 1 Department of Genetics and Plant Breeding, Banda University of Agriculture and Technology, Banda 210 001, India; cmsingh@buat.edu.in (C.M.S.); mukulkumar@buat.edu.in (M.K.); hiteshkumar@buat.edu.in (H.K.); 2 Department of Basic and Social Sciences, Banda University of Agriculture and Technology, Banda 210 001, India; shalinipurwar@buat.edu.in; 3 Department of Plant Protection, Banda University of Agriculture and Technology, Banda 210 001, India; akhileshkumarsingh@buat.edu.in; 4 Department of Entomology, Banda University of Agriculture and Technology, Banda 210 001, India; bksingh@buat.edu.in; 5 Crop Improvement Division, ICAR-Indian Institute of Pulses Research, Kanpur 208 024, India; aditya.pratap@icar.gov.in; 6 Department of Biotechnology, Yeungnam University, Gyeongsan 38541, Republic of Korea

**Keywords:** seedling waterlogging, crop wild relatives, ARF, auxin-encoding genes, *Vigna umbellata*

## Abstract

Mungbean is known to be susceptible to waterlogging (WL) stress. Some of the wild species have the potential to tolerate this through various physiological and molecular mechanisms. *Auxin Response Factor* (ARF) and *Auxin/Indole Acetic Acid* (AUX/IAA), an early responsive gene family, has multiple functions in growth, development, and stress tolerance. Here, we report the first comprehensive analysis of the ARF and AUX/IAA gene family in mungbean. A total of 26 ARF and 19 AUX/IAA genes were identified from the mungbean genome. The ARF and AUX/IAA candidates were clearly grouped into two major clades. Further, the subgrouping within the major clades indicated the presence of significant diversity. The gene structure, motif analysis, and protein characterization provided the clue for further fundamental research. Out of the10 selected candidate genes, *VrARF-5*, *VrARF-11*, *VrARF-25*, and *VrAUX/IAA-9* were found to significantly multiple-fold gene expression in the hypocotyl region of WL-tolerant wild relatives (PRR 2008-2) provides new insight into a role in the induction of lateral root formation under WL stress. The analysis provides an insight into the structural diversity of ARF and AUX/IAA genes in mungbean. These results increase our understanding of ARF and AUX/IAA genes and therefore offer robust information for functional investigations, which can be taken up in the future and will form a foundation for improving tolerance against waterlogging stress.

## 1. Introduction

The genus *Vigna* is known as a diverse group of crops [1]; among them, mungbean is an important warm-season crop. It is a short-duration contingent crop that has high nutritious value including protein and other micro-nutrients. It also improves soil health due to a symbiotic association that improves soil fertility. Considering the acknowledged importance of this crop, its productivity is hampered by various biotic and abiotic stresses [2,3]. However, it is the main crop of the rainy season; therefore, crop growth and productivity are affected by high moisture and waterlogging stress. Waterlogging creates an anaerobic situation and inhibits root development and establishment, which leads to a reduction in the biomass of the plant. The induction of lateral roots is one of the basic adaptive mechanisms to cope with the effect of waterlogging [4]. Auxin has long been recognized as an important phytohormone, which has a diverse role in plant growth and development and stress responses [5,6]. It is widely distributed in higher plants. It has been suggested by earlier findings that the interaction of Aux/IAA with auxin response factor (ARF) has an important function in auxin signaling. The activity of ARF protein is inhibited by dimerization with Aux/IAA under low auxin concentration [7,8]. Enhanced auxin concentration leads to the release of ARF from a repressor heterodimer by promoting the degradation of Aux/IAA proteins through the TIR1 (ubiquitin-proteasome protein) pathway [9]. The ARF, in turn, binds to auxin response elements (AuxREs) on the promoters of these genes and regulates the expression of other primary/early auxin response genes [10]. A typical ARF protein comprises a conserved “N-terminal B3-like DNA-binding domain (DBD)” that regulates the expression of auxin-responsive candidates, a “C-terminal dimerization domain (CTD)” resembling domains III and IV of Aux/IAA proteins, and a variable middle region (MR) [11]. Aux/IAA proteins have been reported to generally have four functional domains, viz., I, II, III, and IV [12]. Domain I, an “N-terminal RD” represented by an “LxLxL” motif [13], can interact with the TOPLESS co-repressor. Domains III and IV, the C-terminal domains, repress the function of ARF and subsequently repress auxin signaling transduction through the dimerization of ARF [14,15,16]. Functional identification and characterization of ARF genes revealed that these genes have important functions in plant developmental stages. In *Arabidopsis*, leaf senescence and floral organ abscission were reported to be regulated by *AtARF1* and *AtARF2* [17], whereas *AtARF2* was reported to act as a transcriptional repressor involved in the auxin-mediated control of leaf longevity [18]. Another gene, *IAA28,* was reported as a promoter of lateral root initiation in response to auxin signals as a transcription repressor [19]. Furthermore, two genes, *NPH4/ARF7* and *ARF19,* were involved in leaf expansion and lateral root formation by auxin induction [20]. In rice, *OsARF12* regulates root elongation; in tomato, three ARF genes including *SlARF4*, *SlARF7,* and *SlARF10*, and an Aux/ IAA gene, i.e., *SlIAA9*, were found to be involved in different functions of fruit development [21,22,23]. The above reports indicated that ARF genes are involved in multiple functions related to growth, development, and stress response. The genome-wide analysis indicated that in crop plants such as *Zea mays*, *Arabidopsis thaliana*, *Sorghum bicolor*, *Oryza sativa*, *Glycine max*, *Brassica rapa*, *Vitis vinifera*, *Medicago truncatula*, *Populus trichocarpa*, *Gossypium raimondii*, etc., the ARF and AUX/ IAA are encoded by relatively large gene families [16,24,25,26,27,28,29,30,31]. However, the systematic analysis of auxin-encoding genes in mungbean is still lagging. In light of these facts, the present study was conducted with the objective of the comprehensive analysis of the ARF and AUX/IAA gene family in mungbean and their expression in root tissues under waterlogging stress.

## 2. Results

### 2.1. Identification and Characterization of Auxin-Responsive Proteins in Vigna radiata

A total of 45 auxin-encoding protein sequences, i.e., VrARF (26) and VrAUX/IAA (19), have been identified in the *Vigna radiata* genome. These sequences are designated as VrARF-1 to VrARF-26 and VrAUX/IAA-1 to VrAUX/IAA-19 (Table S1). The lengths of VrARF and VrAUX/IAA proteins exhibited a range from 283 amino acids (VrARF-7) to 1142 amino acids (VrARF-14) in the case of VrARF, and from 183 amino acids (VrAUX/IAA-4) to 880 amino acids (VrAUX/IAA-2) in the case of VrAUX/IAA. Correspondingly, their molecular weights spanned from 31.83 to 126.85 kDa for VrARF and 20.14 to 95.8 kDa for VrAUX/IAA. The isoelectric points (pI) ranged between 5.12 (VrARF-5) and 8.88 (VrARF-26) for VrARF, and from 5.23 (VrAUX/IAA-5) to 8.46 (VrAUX/IAA-8) for VrAUX/IAA. Analysis of the coding sequence (CDS) lengths showed substantial structural diversity, varying from 841 to 4328 bp in VrARF and from 429 to 2813 bp in VrAUX/IAA. Subcellular localization prediction using the WoLF PSORT program indicated that the majority of VrARF and VrAUX/IAA proteins were primarily situated in the outer membrane, extracellular space, and periplasm, as detailed in Table S1.

### 2.2. Evolutionary Tree, Gene Structure, Domain Architectures, and Motif Analyses

**Figure 1:**
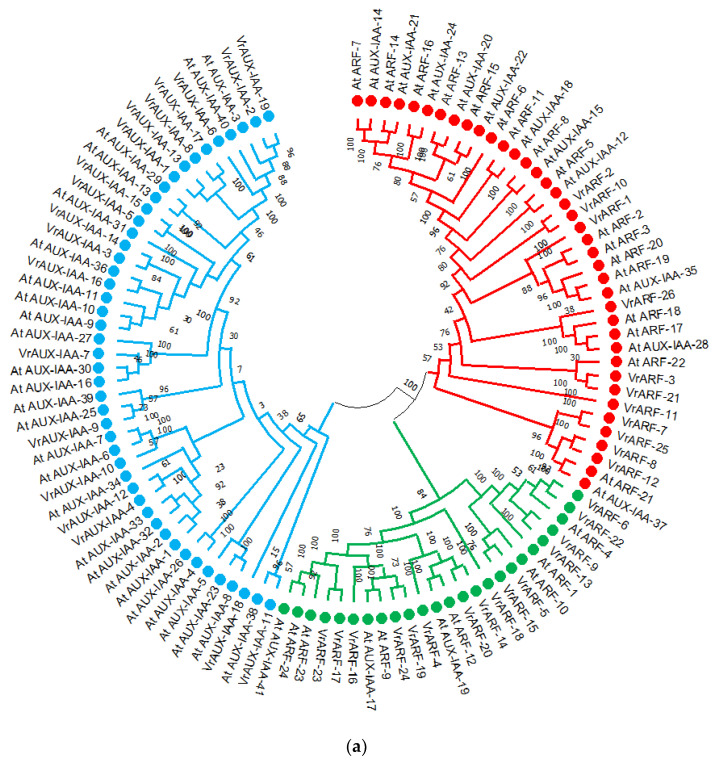

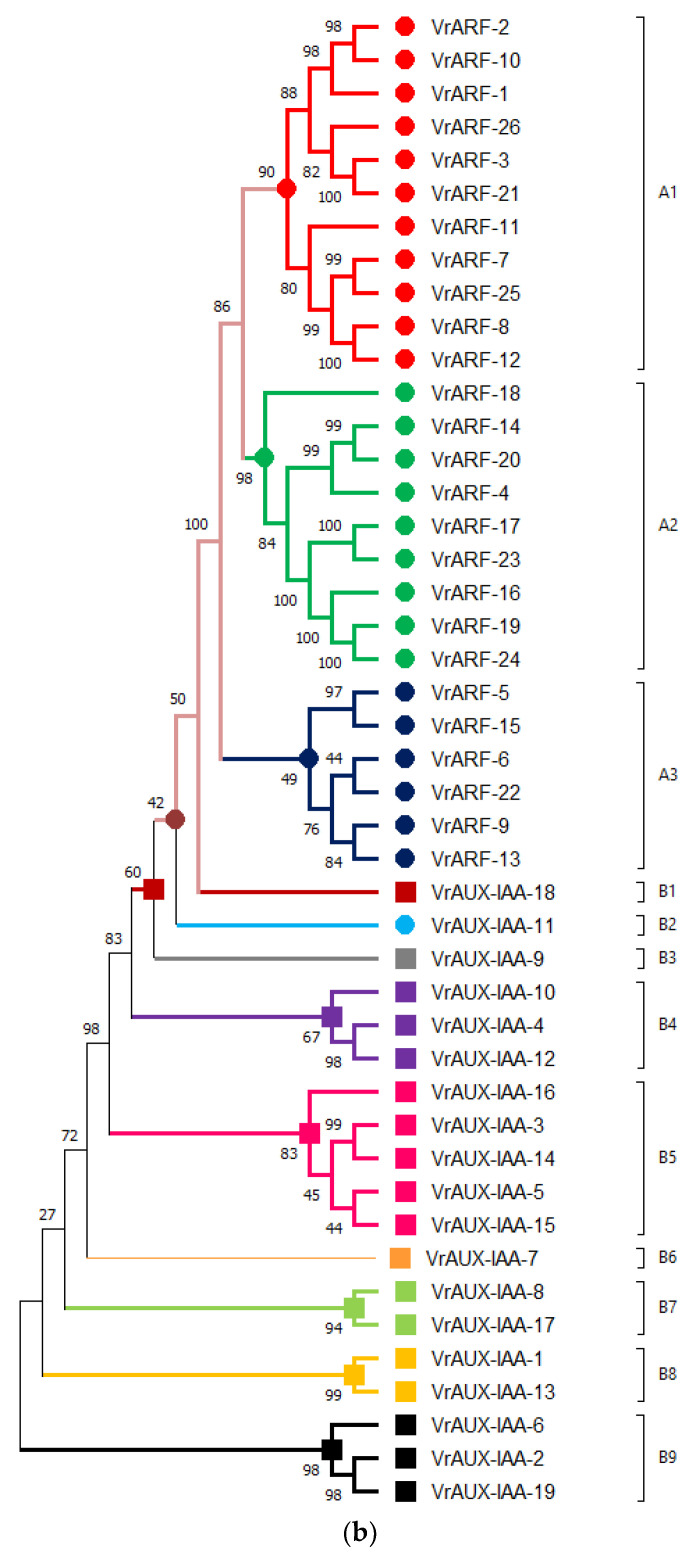
(**a**) Evolutionary tree of *ARF* and *AUX/IAA* genes in *Vigna radiata* and *Arabidopsis thaliana.* The *VrARF* and *VrAUX/IAA* protein sequences from *Vigna radiata* and *Arabidopsis thaliana* were used to construct the tree using MEGA 11 with the ML method. Subgroups 1, 2, and 3 are indicated by red, green, and blue colors, respectively. (**b**) Evolutionary relationship of *VrARF* and *VrAUX/IAA* genes. The *VrARF* and *VrAUX/IAA* protein sequences from *Vigna radiata* were used to construct the tree using MEGA 11 with the ML method.

To investigate the evolutionary relationship of *VrARF* and *VrAUX/IAA* genes, a tree was constructed by including 65 protein sequences of *At_ARF* and *At_AUX/IAA* from *Arabidopsis* (*AtSBT*), as shown in Figure 1a. The evolutionary tree is divided into three major groups. Notably, all three groups contain a mixture of both ARF and ARF-IAA genes. In group 1, there are 26 genes including 4 *VrARF* genes, 9 *AtAUX/IAA* genes, and 13 *AtARF* genes. Group 2 comprises 14 *VrARF* genes, 5 *AtAUX/IAA* genes, and 7 *AtARF* genes. Group III primarily consists of 19 *VrAUX/IAA* genes, 26 *AtAUX/IAA* genes, 4 *VrARF* genes, and 3 *AtARF* genes. It is worth noting that *AtARF* and *VrARF* genes are found in close association with each other, while *AtAUX/IAA* genes and *VrAUX/IAA* genes also tend to cluster together.

Further, the relationship between *VrARF* and *VrAUX/IAA* genes was assessed by constructing an evolutionary tree using 45 protein sequences of both genes (Figure 1b). The *VrARF* genes were categorized into major two subgroups—subgroup-A and subgroup-B, where the VrARF were grouped in one subgroup, while the VrAUX/IAA were grouped into another subgroup. The subgroupings within the major clades were also noted as A1, A2, and A3. In contrast, the *VrAUX/IAA* were grouped into eight subgroups: B1, B2, B3, B4, B5, B6, B7, and B8. The inter and group similarity is represented in Table S2.

**Figure 2:**
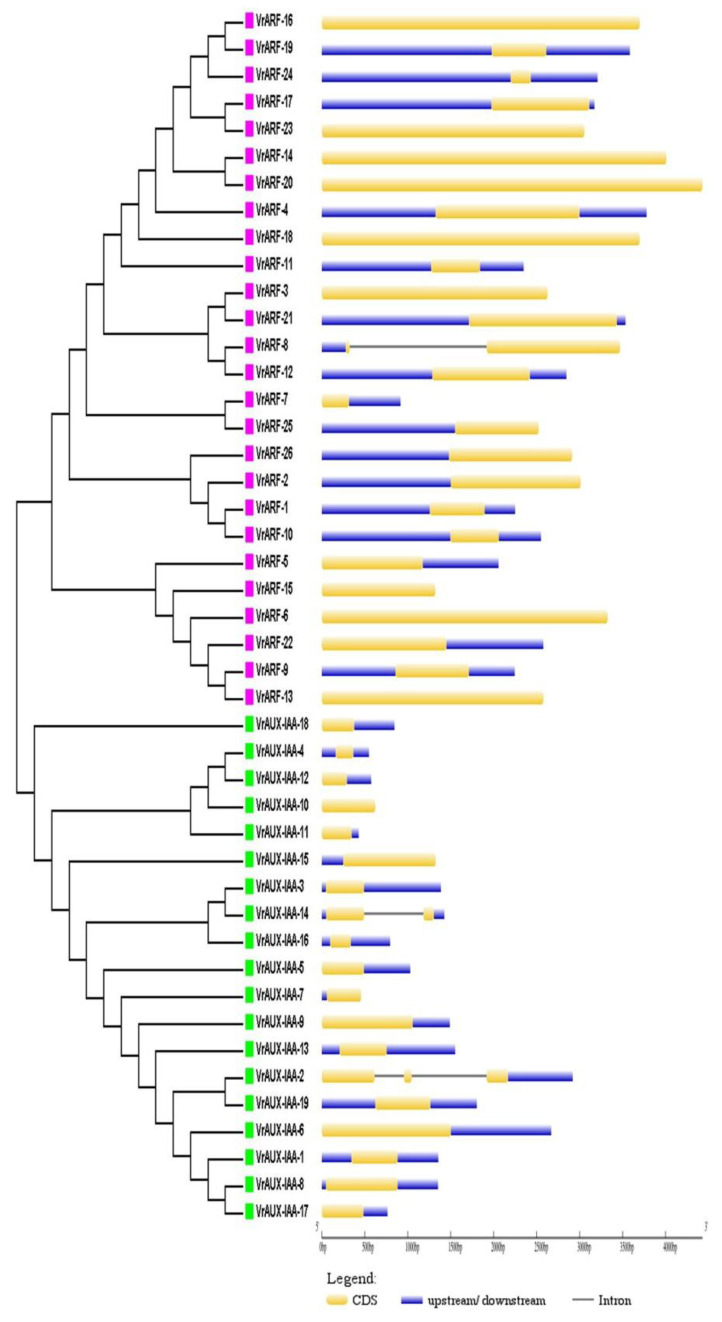
Intron–exon gene structure analysis of *VrARF* and *VrAUX/IAA* genes. The yellow color shows the CDS region, the black line indicates introns, and the blue color indicates up and down regulatory sequences.

**Figure 3:**
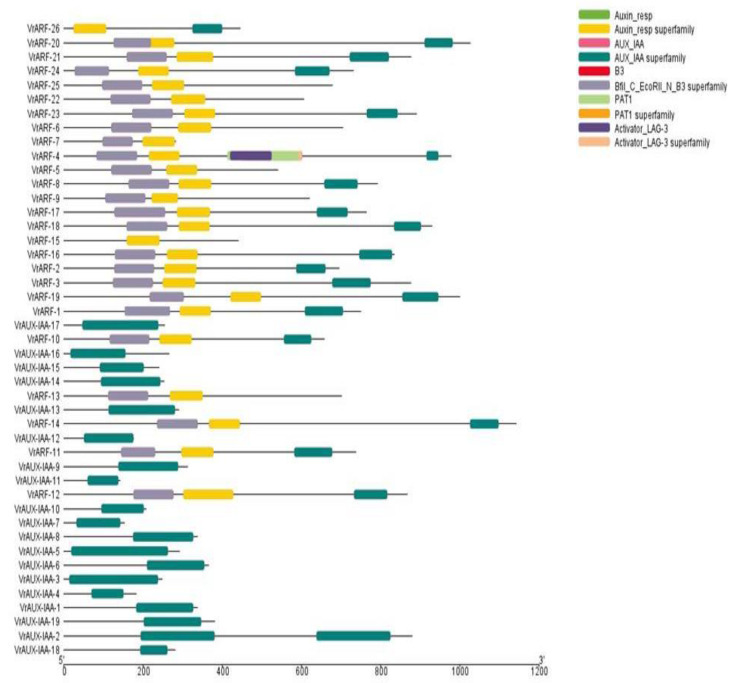
Characterization of VrARF and VrAUX-IAA protein structures by domain distribution.

Regarding gene structure, most of the *VrARF* and *VrAUX/IAA* genes consisted of a single exon, although there were exceptions in *VrAUX/IAA 2* and *VrAUX/IAA 14*, which contained two and three exons, respectively. While the genes within the same subgroup exhibited similar gene structures and exon numbers, variability in intron length was observed. This conservation in gene structure within each *VrARF* and *VrAUX/IAA* group underscored their high sequence similarity (Figure 2). Analysis of the domain architectures (Figure 3) revealed that both *VrARF* and *VrAUX/IAA* shared the *Auxin_IAA*, also known as the CTD superfamily domain, at the C-terminal. Additionally, *VrARF* carried supplementary domains such as the *Auxin_resp*, known as an MR superfamily, at the middle region, and the *N-terminal B3 domain* superfamily known as DBD (Bfill_c_EcoRII_N_B3 superfamily). It shows that all *VrARFs/VrAUX/IAA* contained the Auxin_response superfamily as an intermediate domain. All VrARF proteins contained a complete Auxin_IAA structure except class II of *VrARFs,* like *VrARF5*, *VrARF6*, *VrARF7*, *VrARF9*, *VrARF13*, *VrARF22*, and *VrARF25*. Similarly, DBD existed in all VrARF proteins except *VrARF26* and *VrARF15*. The “B3 domain superfamily” is known as the housing DNA-binding domain found in various transcription factors (TFs) and regulatory proteins (RPs). These domains play pivotal roles in DNA binding and gene expression regulation controlling diverse cellular processes like development, stress responses, and signaling pathways. Notably, VrARF 4 featured the PAT1 domain, implicated in mRNA translation and degradation regulation, and often linked with cytoplasmic P-bodies involved in mRNA storage, degradation, and translational repression.

**Figure 4:**
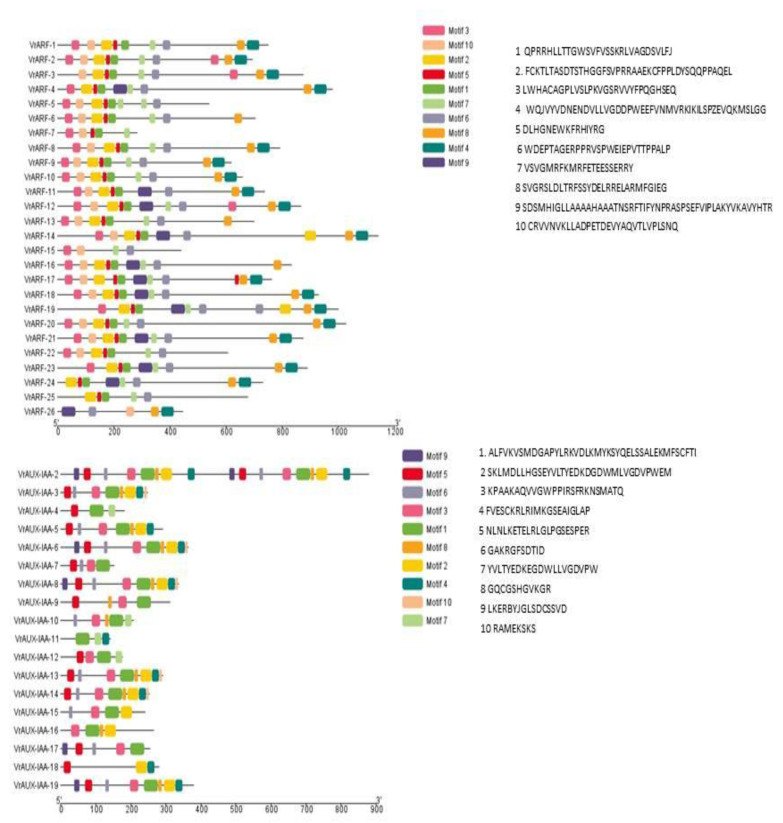
Characterization of VrARF and VrAUX-IAA protein structures by protein motif distribution.

The basic units of protein structures are protein motifs that directly determine the function of given proteins (Figure 4). To explain the diversification of VrARF and VrAUX/IAA proteins, the conserved and diverged motifs were further identified. A typical *Aux/IAA* gene is composed of four structure motifs (I, II, III, and IVs) [32]. Most of the *VrARF/VrAUX/IAA* members contained four motifs. Seven members (VrARF5, VrARF6, VrARF7, VrARF9, VrARF13, VrARF22, and VrARF25) contained three motifs, while two members (*VrARF26* and *VrARF15*) contained only two domains. In the *VrARF/VrAUX/IAA*, a total of 10 conserved motifs were identified. The B3 domain corresponded to motifs 1, 2, and 5; likewise, the *ARF* domain consisted of motifs 6, 7, and 9; and the CTD domain was formed by motifs 4 and 8. Furthermore, the B3 domain and the *ARF* domain constituted a conserved DBD structure. There were 17 *ARF* members (*VrARF 1*, *2*, *3*, *4*, *8*, *10*, *11*, *12*, *14*, *16*, *17*, *18*, *19*, *20*, *21*, and *23*), which contained all of the three domains, whereas *VrARF 15* contained only the ARF domain, and the rest of the 19 *VrAUX/IAA* contained only the CTD domain.

### 2.3. Chromosome Distribution and Synteny Analysis

**Figure 5:**
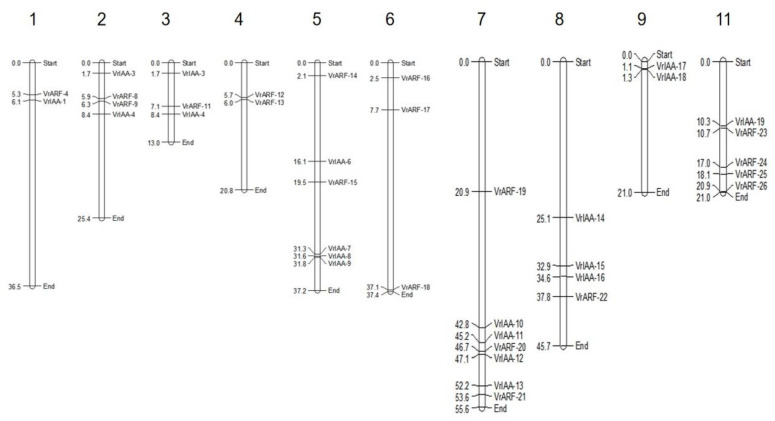
Chromosomal distribution of *ARF* and *AUX/IAA* genes on mungbean (*V. radiata*) genome. The chromosome size is indicated by its relative length using the information from LIS. The scale (in megabase) on the left depicts the relative lengths of the different chromosomes in mungbean. The genes are depicted on the right side of each chromosome corresponding to the position of each gene. The linkage groups are given on the top. The figure was constructed using the MapChart program v 2.32.

A total of 26 *VrARF* and 19 *VrAUX/IAA* genes were identified in the mungbean genome (Figure 5). Among these, seven *VrARF* genes were situated on specific scaffolds, while two scaffolds contained *VrAUX/IAA* genes. The remaining *VrARF* and *VrAUX/IAA* genes were mapped onto different chromosomes. Notably, all the chromosomes carried *VrARF/VrAUX/IAA* genes except for chromosomes 4 and 6, which exclusively harbored *VrARF* genes, and chromosomes 9 and 11, which lacked VrARF genes. Chromosome 7 stood out with the highest number (7) of VrARF/VrAUX/IAA genes, whereas chromosome 1 had the fewest (2) genes.

**Figure 6:**
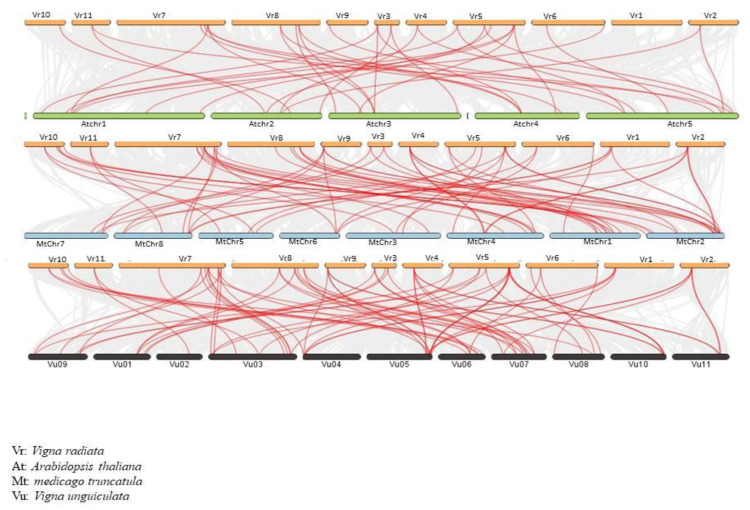
Synteny analysis of IAA and ARF genes in the genomes of *Medicago truncatula*, *Arabidopsis thaliana*, and *V. ungiculata*. Orange, green, blue, and black marks represent the *V. radiata*, *Arabidopsis thaliana*, *Medicago trucatula*, and *Vigna ungiculata* chromosomes.

To understand the synteny of *VrARF/VrAUX/IAA* genes across genomes, we conducted an analysis involving *Arabidopsis thaliana*, *M. truncatula*, and *Vigna unguiculata* (Figure 6 and Tables S3–S5). We compared the entire genomes of *V. radiata* and *Arabidopsis thaliana*, resulting in 34 gene pairs exhibiting synteny. Impressively, more than 60 gene pairs displayed collinear relationships between *V. radiata* and *M. truncatula*, and around 70 gene pairs were noted between *V. radiata* and *Vigna unguiculata*. The complexity of collinear relationships between *V. radiata* and *V. unguiculata*, as well as *M. truncatula*, surpassed that observed between *M. truncatula* and *Arabidopsis thaliana.* These findings collectively imply that *V. radiata* and *Arabidopsis thaliana* are relatively distantly related, while they show closer affinities with *M. truncatula* and *V. unguiculata*. Furthermore, within the same gene family, *V. radiata* genes share a closer relationship with those of *V. unguiculata* compared to *Arabidopsis thaliana*.

### 2.4. Prediction of VrARF and VrAUX/IAA Family Interaction Networks

**Figure 7:**
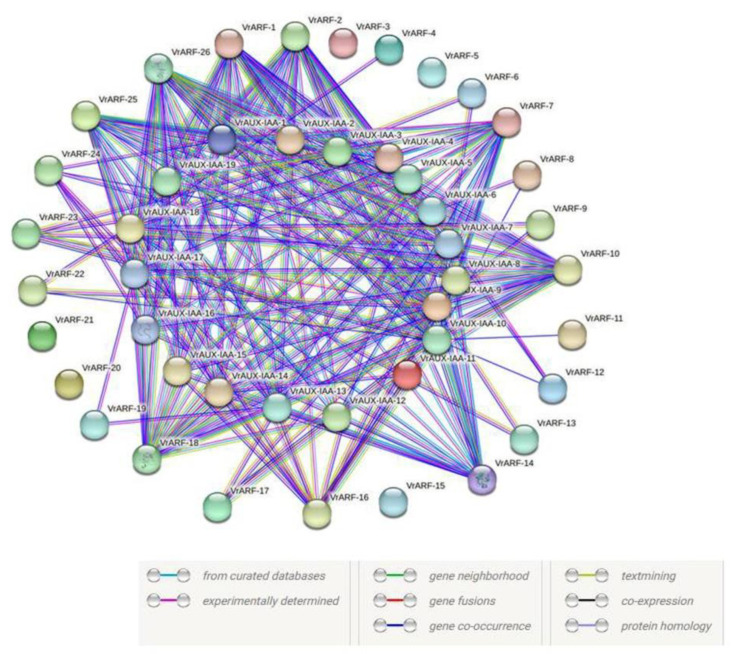
Predicted protein–protein interaction network of *VrARFs* and *VrAUX/IAA.* The network contains 39 nodes (18 *VrAUX/IAAs* and *21 VrARFs*).

Using the STRING (https://string-db.org/) software [33] (accessed on 5 September 2023), we predicted a protein–protein interaction network between VrARF and VrAUX/IAA. This network revealed interactions among 18 VrAUX/IAAs and 21 VrARFs forming a complex web of protein interactions (Figure 7). Our findings indicated that certain VrARF proteins had the ability to interact with multiple VrAUX/IAAs, and conversely, some VrAUX/IAAs exhibited interactions with multiple VrARFs. Notably, we observed that eight VrARF proteins (VrARF-1, VrARF-2, VrARF-7, VrARF-10, VrARF-14, VrARF-18, VrARF-25, and VrARF-26) which function as activators, displayed strong interactions with a majority of VrAUX/IAA proteins. Additionally, we found that VrAUX/IAA-11 interacted not only with VrAUX/IAA-14 but also with several VrARFs, indicating its central role in mediating interactions within this network. Moreover, we identified co-expression correlations among many VrARF genes, suggesting their involvement in shared regulatory pathways. For instance, VrAUX/IAA-9 displayed high co-expression levels with 15 VrARFs, as well as with VrAUX/IAA-2. Similarly, VrAUX/IAA-8 exhibited co-expression with 10 VrARFs. These findings suggest that VrAUX/IAA-9, VrAUX/IAA-2, and VrAUX/IAA-8 might serve as key regulators among the 45 VrARFs present in this network. This predictive protein–protein interaction network and the co-expression patterns uncovered in our study offer valuable insights into the intricate regulatory mechanisms involving VrAUX/IAA and VrARF proteins, shedding light on their potential roles in various cellular processes.

### 2.5. Expression Profiling

**Figure 8:**
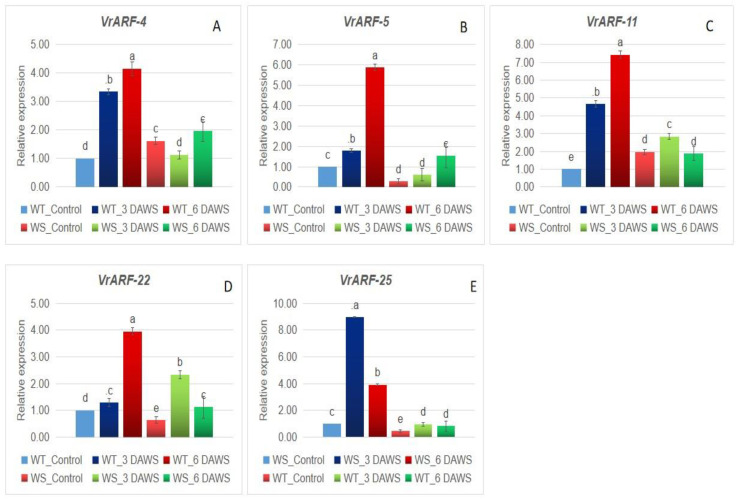
qRT-PCR analysis of selected *VrARF* candidate genes (**A**) *VrARF-4,* (**B**) *VrARF-5,* (**C**) *VrARF-11,* (**D**) *VrARF-22,* (**E**) *VrARF-25* on WT-*V. umbellata* (PRR 2008-2) and WS-.*V. radiata* (IPM 2-3) under waterlogging stress. The same letter shows non-significant differences, whereas different letters show significant differences.

**Figure 9:**
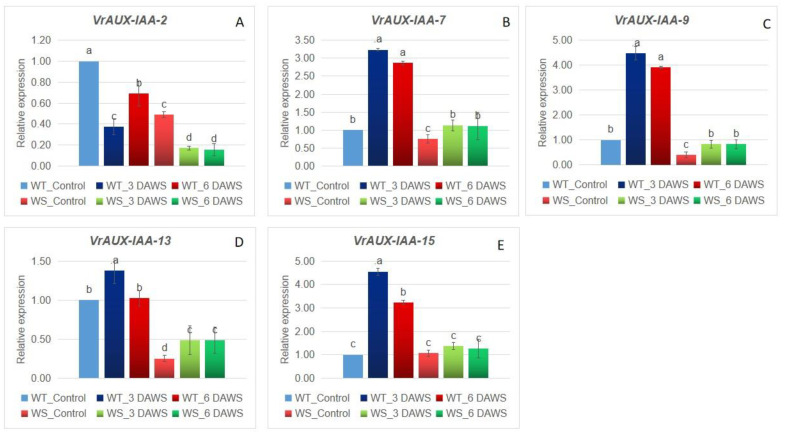
qRT-PCR analysis of selected *VrAUX/IAA* candidate genes (**A**) *VrAUX/IAA-2,* (**B**) *VrAUX/IAA-7,* (**C**) *VrAUX/IAA-9,* (**D**) *VrUX/IAA-13,* (**E**) *VrAUX/IAA-15* on WT-*V. umbellata* (PRR 2008-2) and WS-.*V. radiata* (IPM 2-3) under waterlogging stress. The same letter shows non-significant differences, whereas different letters show significant differences.

Five *VrARF* and five *VrAUX/IAA* genes selected based on the presence of different domains were used for expression profiling in the hypocotyl region of one waterlogging-susceptible (WS), IPM 2-3, and waterlogging-tolerant (WT), PRR 2008-2 (Figure 8 and Figure 9). The *VrARF-1* exhibited a significant increase (about 3.2-fold) in the WT genotype 3 days after waterlogging stress (3-DAWS), whereas it was upregulated about 4.1-fold at 6 DAWS. In WS, it was upregulated but expression was very low, about 1.2–1.8-fold only. A similar pattern was noticed with the VrARF-5, 11, and 22, but the extent of expression was higher at 6 DAWS, at about 5.9-, 7.1-, and 3.9-fold, respectively. The candidate gene *VrARF-25* showed the maximum expression of about 83-fold at 3 DAWS in the WT genotype with very lower up-regulation in WS. The relative expression of *VrAUX/IAA-2* was found to decrease at all the day points under waterlogging stress in both the WS and WT genotypes. *VrAUX/IAA-7*, *VrAUX/IAA-9,* and *VrAUX/IAA-15* were found upregulated in WT genotypes upon waterlogging stress, whereas a significant decrease was noticed in the WS genotype upon waterlogging stress.

## 3. Discussion

Auxin, a plant hormone, is crucial for controlling different aspects of growth and development in plants. Two important components in auxin signaling are IAA and ARF, which play important roles in regulating downstream processes [34]. The quantities of IAA and ARF components vary among different plant species. For instance, *Arabidopsis thaliana* contains 29 *AtIAAs* and 23 *AtARFs*, rice contains 31 *AtIAAs* and 25 *AtARFs*, *M. truncatula* contains 25 *MtIAAs* and 40 *MtARFs*, and soybean contains 63 *GmIAAs* and 55 *GmARFs* [16,35,36,37,38,39]. In the present study, we focused on *V. radiata* and identified 26 *VrARF* genes and 19 *VrAUX/IAA* genes using updated genome data. These genes exhibited variations in their predicted molecular weight (MW) and isoelectric point (pI), similar to observations in rice IAAs and *Brachypodium distachyon* ARF [16,40]. These variations suggest that the diverse *VrARF/VrAUX/IAA* proteins might serve different functions based on their microenvironment. During the evolutionary processes of genes, changes have been made in their structure, which has led to the addition or reduction of exons/introns. In addition, it was reported that the induction speed of a gene is influenced by the intron number, and genes with fewer introns might be quickly induced [41,42,43]. Interestingly, all *VrARF* and *VrAUX/IAA* genes consist of a single exon. This is distinct from some other plants like *Brachypodium distachyon*, *Oryza sativa*, and *Prunus mume*, which have multiple exons. In cases where genes within the same subgroup have a similar number of exons and introns, it often suggests similar functions. However, exceptions have been noted in *Arabidopsis*, chickpeas, rice, and tobacco, where the number of exons and introns differs despite being in the same subfamily [37,44]. This variation in gene structure within a subfamily may arise from the genes evolving diverse functions over time. An Aux/IAA protein usually comprises four specific sections named I, II, III, and IV, known as domains [9,45]. Among the total of 26 *VrARF* and 19 *VrAUX/IAA* proteins, most contained all four domains. However, some proteins, such as *VrARF5*, *VrARF6*, *VrARF7*, *VrARF9*, *VrARF13*, *VrARF22*, and *VrARF25*, were missing at least one domain. The absence of domain I in proteins like 7 suggested a possible loss in their ability to engage TOPLESS (TPL) co-repressors. Consequently, they might lose their function as repressors in auxin signaling. Moreover, proteins like *VrARF26* and *VrARF15*, along with their counterparts in *Arabidopsis thaliana* [34], lacked domain II. This indicated that these proteins wouldn’t degrade under higher levels of auxin. Recent research has revealed that, instead of TIR1/AFB-mediated degradation, non-canonical Aux/IAA proteins are stabilized by auxin-triggered phosphorylation via upstream protein kinases. This stabilization plays a role in gene expression through ARF transcription factors, which in turn influences differential growth during certain developmental processes. It is also possible that some of the deduced protein sequences could be pseudogenes or have low expression levels. The expression of genes without domain II was notably low in root tissues, which aligned with findings in other plant species like *Brassica napus*. Similarly, certain *VrIAA* genes, such as *VrAUX/IAA26* and *VrAUX/IAA15*, had lost both domains I and II. As observed in other plants, various truncated Aux/IAA proteins exist, each associated with distinct functions within the auxin signaling pathway. To put it simply, Aux/IAA proteins are composed of four segments. Some VrARF and VrAUX/IAA proteins lack certain segments, affecting their role in auxin signaling. These variations in segments are linked to the different functions that Aux/IAA proteins perform in the auxin signaling process across various plants.

An ARF protein typically consists of three distinct sections: DBD, MR, and CTD [46]. These components serve specific functions within the protein. The DBD is responsible for binding to auxin response elements located in the promoters of genes responsive to auxin signals [47]. The MR region’s amino acid composition determines whether it acts as an activator or repressor [26]. On the other hand, the CTD takes part in interactions between different ARF proteins [48]. Among the VrARF proteins, 15 have complete domains, whereas *VrARF15* is missing both the DBD and CTD domains. The rest of the VrARF proteins lack only the CTD domain. This observation suggests that some VrARF proteins may operate in a manner not dependent on auxin signals. Similar to this, *AtARF3*, which is without the CTD domain, doesn’t interact with elements of the standard TIR1/AFB signaling pathway. Instead, it functions independently of the TIR1/AFB receptor [49]. We conducted a synteny analysis of IAA and ARF genes in various plant species including *V. radiata*, *M. truncatula*, *A. thaliana*, and *Vigna unguiculata* using the TBtools software (https://github.com/CJ-Chen/TBtools/releases accessed on 5 September 2023). This analysis revealed a total of 34 instances of shared *VrARF/VrAUX/IAA*-*AtARF/IAA* genes within the Aux/IAA family. Furthermore, we identified 60 pairs of *VrARF/VrAUX/IAA* and *MtARF/IAA* genes, as well as 73 pairs of *VrARF/VrAUX/IAA* and *VuARF/IAA* genes within the ARF family. These findings strengthen the notion that *V. radiata* is more closely related to *V. unguiculata* and *Medicago truncatula ARF/AUX/IAA* genes than to *Arabidopsis thaliana* in terms of evolutionary relationships.

Protein–protein interactions play a pivotal role in numerous biological processes, including signal transduction, as well as the regulation of gene expression. One critical aspect of these interactions involves the mediation of auxin responses through the interplay between ARF (Auxin Response Factor) and Aux/IAA proteins [13]. Therefore, investigating the interaction between IAA and ARF in *V. radiata* has significant importance. In our study, we constructed protein–protein interaction networks that unveiled 192 unique interaction combinations involving 18 *VrAUX/IAA* and 21 *VrARFs*. In contrast, we observed 161 specific interactions among 18 *MtIAAs* and 24 *MtARFs* in *M. truncatula*, whereas *Arabidopsis thaliana* displayed a large number (213) of specific interactions involving 19 ARFs and 29 *Aux/IAAs*. Remarkably, our data highlighted that up to 84% of ARFs interacted with Aux/IAA factors, a finding substantiated by the successful integration of the co-expression maps with the protein–protein interaction data [50]. Additionally, our observations illustrated that a single ARF protein in *V. radiata* could interact with multiple Aux/IAA proteins, and conversely, the reverse also held true. For instance, VrARF 1, 2, 14, 18, 25, and 26 exhibited interactions with 18 *VrAUX/IAA*. *VrAUX/IAA-11* exhibited interactions not only with *VrARF-16* but also with *VrAUX/IAA-16* proteins. This pattern of interactions parallels the findings in tomato plants, where *SlARF2A* has been reported to interact with five SlIAAs, and *SlARF6A* with at least 11 *SlIAAs* [51]. Likewise, in *M. truncatula*, MtARF29 displayed interactions with *MtIAA12* and *MtIAA21*, along with numerous *MtARFs* [32]. Similarly, key transcriptional activators such as *AtARF5*, *AtARF6*, *AtARF7*, *AtARF8*, and *AtARF19* were observed to interact with almost all the Aux/IAA proteins in the case of *Arabidopsis thaliana* [50]. The predictive interaction networks, as well as the co-expression network generated through our study, offer valuable insights for further exploration into the regulation of *VrAux/IAA-VrARF* interactions and their influence on growth and development, in addition to an adaptation to environmental stresses in *V. radiata*.

Crop wild relatives (CWRs) are known for their potential for stress tolerance against various biotic and abiotic stresses [2,3,52,53]. Kumari et al. [54] explored the endemic wild *Vigna* species and identified some of the potential sources of resistance to yellow mosaic disease. Sahu et al. [55] screened the cultivated wild *Vigna* gene pool and identified potential sources of bruchid resistance. Purwar et al. [56] performed the expression of R genes in two wild *Vigna* species, and their comparative study with mungbean, and found the higher expression in wild species. Likewise, Tripathi et al. [57] noticed a higher basal and induced expression of four R gene wild two *Vigna* accessions. These indicated the potential of wild species. The expression analysis of WS and WT genotypes indicated that the *VrARF-5*, *VrARF-11*, *VrARF-25*, *VrAUX/IAA-9,* and *VrAUX/IAA-15* play an important role in waterlogging stress tolerance in the WT *Vigna* genotype, PRR 2008-2. Therefore, the results of the present study pave the way for further investigation on the functional characterization of ARF and AUX/IAA proteins and their further potential use toward genetic amelioration of mungbean for waterlogging stress tolerance.

## 4. Materials and Methods

### 4.1. Identification and Characterization of VrARF and VrAUX/IAA Protein Sequences

The Hidden Markov Model (HMM) of VrARF and VrAUX/IAA was obtained from the Pfam PF06507 and PF 02309 database as a query to search against the *Vigna radiata* genome database of the legume information system (https://www.legumeinfo.org/ (accessed on 1 June 2023). Further, each of the selected candidates was examined for the conserved structure domain using NCBI-CDD [58]. ExPAsy was used to estimate the molecular weight (MW) and isoelectric points (pI) of the *MaSBTs* [59]. The sub-cellular localization and signal peptide were predicted by using the online software CELLO life, WoLF PSort (http://cello.life.nctu.edu.tw/ (accessed on 5 September 2023), https://wolfpsort.hgc.jp/ (accessed on 5 September 2023)), and SignalP (https://services.healthtech.dtu.dk/service.php?SignalP-5.0 (accessed on 5 September 2023), respectively [60,61,62].

### 4.2. Evolutionary Tree, Domain, Gene Structure and Motif Analyses

The identified VrARF and VrAUX/IAA protein sequences were aligned using Clustal Omega with the default parameters. The evolutionary trees were prepared by using MEGA 11 using the maximum likelihood (ML) method based on the Jones–Taylor–Thornton (JTT) matrix-based model > 1000 replications bootstrap [63]. *VrARF* and *VrAUX/IAA* genes were checked for intron and exon structure using GSDS software V. 2.0. The gene structure characteristics and exon–intron organizations of the VrARF and VrAUX/IAA were exhibited using the TBtools program, based on the comparison among the full-length genome sequences and the protein-coding sequences of the given genes [64]. The VrARF (26) and VrAUX/IAA (19) protein domains and active motif functions were analyzed in the Pfam database (pfan.xfam.org). The web-based motif identification servers, MEME-Suit (Multiple Em for Motif Elicitation, meme-suite.org/meme/), were used to detect potential motifs with the following parameters: motif width < 50, motifs < 20, and e-value < 1 × 10^−5^ [65].

### 4.3. Chromosomal Distribution and Synteny Analyses

The physical position of all the identified *VrARF* and *VrAUX*/*IAA* genes were obtained from the legume information system (https://www.legumeinfo.org/ accessed on 5 September 2023) and genes from short arm to long arm were mapped on to their corresponding chromosomes in ascending order using MapChart 2.32 (http://www.joinmap.nl accessed on 5 September 2023). The syntenic blocks of *VrARF* and *VrAUX/IAA*, *AtARF /AUX/IAA*, *MtARF/IAA*, and *VuARF/IAA* were visualized by Mcscan [66].

### 4.4. Plant Materials and Stress Treatment

The plant material for the present study comprised one waterlogging-susceptible (WS, IPM 2-3) and one waterlogging-tolerant (WT, PRR 2008-2) *Vigna* accessions belonging to *V. radiata* and *V. umbellata*. Two sets of experiments were conducted under a non-stressed environment (control) and a stressed environment (WL). Each set of experiments was laid out in a completely randomized block design (CRD) with three replications. Ten seeds from each accession were grown in seedling bags. The 7-day-old seedlings were subjected to WL (2 cm WL from the soil surface) for up to 6 days.

### 4.5. RNA Extraction and cDNA Synthesis

The 100 mg of frozen hypocotyl samples in the liquid nitrogen were homogenized using tissue lyser-II (Qiagen, Hilden, Germany). This was followed by RNA extraction using the plant RNA extraction kit (RNeasy Mini Kit, Qiagen) as per the manufacturer’s instructions. Subsequently, the RNA was subjected to DNase treatment to remove the DNA contaminants, and 1 µg of RNA was reverse-transcribed by using the Revert Aid First Strand cDNA Synthesis Kit (Thermo Fisher Scientific, Waltham, MA, USA). The quantification of cDNA was conducted on a micro-volume spectrophotometer (QIAExpert, Qiagen) and normalized by 100 ng/µL for qRT-PCR analysis.

### 4.6. Quantitative Real-Time PCR Analysis

Ten gene-specific primers were designed for expression analysis (Table S6). The PCR reactions comprised 10 µL of 2 × SYBR green q-PCR master mix (Thermo Fisher Scientific), 1 µL of 10 pmol each of forward and reverse primers (Eurofins, India), 6 µL of nuclease-free water, and 2 µL of cDNA. The fast cycling approach was adopted, with 2 min initial denaturation at 96 °C, 40 cycles of 20 s denaturation at 96 °C, and 45 s annealing and extension at 60 °C. The *Actin* gene was used as an internal control. qRT-PCR analysis was carried out using a Rotor Gene Q-6000 RealTime PCR machine (Qiagen). Three biological replicates were taken, and two technical replicates were used for expression analysis. The relative expression levels of the genes were calculated via the delta-delta CT method [67].

### 4.7. Predicted Protein Interaction Network and Co-Expression Network Construction

The interacting networks of VrARF and VrAUX/IAA proteins were integrated into the STRING [68] (https://www.string-db.org/ (accessed on 5 September 2023) software, followed by an export of the co-expression network data from STRING, which was further calculated using Microsoft Excel 2019.

## Data Availability

All the data generated in this experiment were presented in the manuscript and its Supplementary Files.

## Supplementary Materials

The following supporting information can be downloaded at: https://www.mdpi.com/article/10.3390/plants12223858/s1, Table S1. Characterization of *VrARF* and *VrAUX/IAA* candidates identified in mungbean (*Vigna radiata*) genome. Table S2: Estimates of average evolutionary divergence over sequence pairs within (intra) and between (inter) the groups. Table S3: Synteny analysis of VrAUX-IAA and VrARF genes with *Vigna radiata* genome. Table S4: Synteny analysis of VrAUX-IAA and VrARF genes with *Medicago truncatula.* Table S5: Synteny analysis of VrAUX-IAA and VrARF genes with *Arabidopsis thaliana.* Table S6: Primers used in the expression profiling.
