# Supplementary material for: Analysis of Auxin-Encoding Gene Family in Vigna radiata and It’s Cross-Species Expression Modulating Waterlogging Tolerance in Wild Vigna umbellata
Source: Plants (Basel). 2023 Nov 15;12(22):3858. doi: 10.3390/plants12223858 (PMC10674698; doi:10.3390/plants12223858)
Supplement: Supplementary file 1 [file plants-12-03858-s001.zip › Table S1.pdf]

Table S1. Characterization of *VrARF* and *VrAUX/IAA* candidates identified in mungbean (*Vigna radiata*) genome.

Alias Name	Gene ID	Chromosomal position	Start	End	CDS length	Number of exons	Protein length	pI	MW
VrARF-1	vigra.Vradi0083s01070	vigra.scaffold_83	349327	353746	2250	1	750	6.39	83.19
VrARF-2	vigra.Vradi0133s00220	vigra.scaffold_133	635503	640092	2654	3	695	5.72	77.19
VrARF-3	vigra.Vradi0185s00050	vigra.scaffold_185	526912	533836	2585	1	876	6.74	97.43
VrARF-4	vigra.Vradi01g03030	1	5279168	5285358	3734	1	979	6.7	107.85
VrARF-5	vigra.Vradi0269s00010	vigra.scaffold_269	304231	308360	1939	1	541	6.51	59.59
VrARF-6	vigra.Vradi0279s00040	vigra.scaffold_279	102138	105984	3207	1	705	7.34	77.89
VrARF-7	vigra.Vradi0284s00060	vigra.scaffold_284	58958	61866	841	1	283	8.72	31.83
VrARF-8	vigra.Vradi02g05690	2	5860942	5867067	3334	2	792	6.24	87.9
VrARF-9	vigra.Vradi02g05890	2	6268170	6271161	2215	1	620	7.82	68.58
VrARF-10	vigra.Vradi0388s00010	vigra.scaffold_388	150501	155095	2269	1	658	6.36	73.64
VrARF-11	vigra.Vradi03g05610	3	7125485	7133596	2279	1	738	7.73	82.2
VrARF-12	vigra.Vradi04g02680	4	5717464	5723221	2836	1	867	6.93	96.31
VrARF-13	vigra.Vradi04g02860	4	5991491	5994639	2554	1	701	7.68	77.21
VrARF-14	vigra.Vradi05g01790	5	2123631	2131173	4005	2	1142	6.51	126.85
VrARF-15	vigra.Vradi05g10740	5	19541651	19544763	1307	1	441	6.67	48.84
VrARF-16	vigra.Vradi06g02570	6	2530143	2537527	2781	1	835	6.51	92.3
VrARF-17	vigra.Vradi06g06060	6	7661994	7673694	3002	1	764	6.51	84.7
VrARF-18	vigra.Vradi06g16730	6	37083558	37088796	3569	1	930	5.44	102.9
VrARF-19	vigra.Vradi07g08370	7	20941116	20952965	3577	1	1000	6.79	110.17
VrARF-20	vigra.Vradi07g23560	7	46650997	46658251	4328	1	1027	6.13	114.34
VrARF-21	vigra.Vradi07g29890	7	53637291	53642315	3409	1	876	6.86	97.68
VrARF-22	vigra.Vradi08g16880	8	37773598	37776940	2200	1	607	7.45	66.51

VrARF-23	vigra.Vradi10g04300	10	10705507	10712363	3033	1	890	5.96	98.43
VrARF-24	vigra.Vradi10g09490	10	17030133	17036561	3207	1	731	6.51	80.56
VrARF-25	vigra.Vradi10g10520	10	18142782	18147934	2435	1	678	8.88	75.55
VrARF-26	vigra.Vradi10g13540	10	20892628	20898729	2633	1	446	6.26	49.39
Vr-AUX/IAA-1	vigra.Vradi01g03820	1	6068351	6070969	1208	1	337	7.73	36.34
VrAUX/IAA-2	vigra.Vradi0270s00020	vigra.scaffold_270	356491	371371	2813	1	880	8.42	95.8
VrAUX/IAA-3	vigra.Vradi03g01240	3	1730765	1733813	1350	1	248	8.2	27.41
VrAUX/IAA-4	vigra.Vradi03g06960	3	8406025	8406942	508	1	183	6.21	20.14
VrAUX/IAA-5	vigra.Vradi0443s00040	vigra.scaffold_443	7543	9822	876	1	292	5.23	31.64
VrAUX/IAA-6	vigra.Vradi05g08280	5	16051738	16056749	2528	1	365	6.08	39.75
VrAUX/IAA-7	vigra.Vradi05g20080	5	31282576	31283264	459	1	153	8.21	16.84
VrAUX/IAA-8	vigra.Vradi05g20470	5	31602022	31604430	1011	1	337	8.46	35.78
VrAUX/IAA-9	vigra.Vradi05g20740	5	31825796	31828950	1292	1	312	6.67	33.9
VrAUX/IAA-10	vigra.Vradi07g20430	7	42784001	42784750	624	1	208	6.13	23.82
VrAUX/IAA-11	vigra.Vradi07g22220	7	45149677	45150214	429	1	143	4.46	15.4
VrAUX/IAA-12	vigra.Vradi07g23940	7	47129957	47130765	531	1	177	6.79	19.47
VrAUX/IAA-13	vigra.Vradi07g28440	7	52157418	52159903	1418	1	291	7.27	31.4
VrAUX/IAA-14	vigra.Vradi08g08900	8	25049799	25052266	1268	1	254	5.61	28
VrAUX/IAA-15	vigra.Vradi08g13270	8	32928626	32932386	1313	1	241	6.13	26.44
VrAUX/IAA-16	vigra.Vradi08g14590	8	34550355	34558346	428	1	265	5.94	29.6
VrAUX/IAA-17	vigra.Vradi09g01380	9	1117766	1118919	751	1	255	6.65	27.44
VrAUX/IAA-18	vigra.Vradi09g01590	9	1312662	1314731	833	1	282	8.01	31.51
VrAUX/IAA-19	vigra.Vradi11g09000	11	10322901	10327454	1733	1	381	6.87	44.37
