# Supplementary material for: Analysis of Auxin-Encoding Gene Family in Vigna radiata and It’s Cross-Species Expression Modulating Waterlogging Tolerance in Wild Vigna umbellata
Source: Plants (Basel). 2023 Nov 15;12(22):3858. doi: 10.3390/plants12223858 (PMC10674698; doi:10.3390/plants12223858)
Supplement: Supplementary file 1 [file plants-12-03858-s001.zip › Table S2.pdf]

Table S2: Estimates of average evolutionary divergence over sequence pairs within (intra) and between (inter) the groups

**Intra Group similarity**		**Inter Group Similarity**		
Gene name	Similarity index	Gene name		Similarity index
VrARF	1.58	VrAux-IAA	At ARF	3.66
VrAux-IAA	1.94	VrAux-IAA	VrARF	4.32
At ARF	1.17	At ARF	VrARF	1.44
At Aux-IAA	2.25	VrAux-IAA	At Aux-IAA	2.41
-	-	At ARF	At Aux-IAA	2.62
-	-	VrARF	At Aux-IAA	3.30
