# Supplementary material for: Analysis of Auxin-Encoding Gene Family in Vigna radiata and It’s Cross-Species Expression Modulating Waterlogging Tolerance in Wild Vigna umbellata
Source: Plants (Basel). 2023 Nov 15;12(22):3858. doi: 10.3390/plants12223858 (PMC10674698; doi:10.3390/plants12223858)
Supplement: Supplementary file 1 [file plants-12-03858-s001.zip › Table S3.pdf]

Table S3: Synteny analysis of VrAUX-IAA and VrARF genes with *Vigna radiata* genome

Auxin responsive genes in *Vigna radiata*	Gene ID (Orthologous)	Chromosome number
VrAUX-IAA-6	Vigun09g186900.1.v1.2	Vu09
VrARF-15	Vigun09g171300.1.v1.2	Vu09
VrARF-14	Vigun09g040800.1.v1.2	Vu09
VrARF-20	Vigun09g040800.1.v1.2	Vu09
VrAUX-IAA-10	Vigun09g210300.1.v1.2	Vu09
VrARF-21	Vigun09g109200.1.v1.2	Vu09
VrARF-11	Vigun01g186200.1.v1.2	Vu01
VrAUX-IAA-4	Vigun01g203000.2.v1.2	Vu01
VrAUX-IAA-3	Vigun01g138800.1.v1.2	Vu01
VrAUX-IAA-14	Vigun01g138800.1.v1.2	Vu01
VrAUX-IAA-15	Vigun01g138800.1.v1.2	Vu01
VrAUX-IAA-19	Vigun02g089400.2.v1.2	Vu02
Vr-AUX-IAA-1	Vigun03g022700.1.v1.2	Vu03
VrAUX-IAA-8	Vigun03g022700.1.v1.2	Vu03
VrARF-14	Vigun03g400000.1.v1.2	Vu03
VrAUX-IAA-6	Vigun03g226300.1.v1.2	Vu03
VrAUX-IAA-10	Vigun03g358700.1.v1.2	Vu03
VrAUX-IAA-11	Vigun03g383200.1.v1.2	Vu03
VrARF-20	Vigun03g400000.1.v1.2	Vu03
VrAUX-IAA-12	Vigun03g404600.1.v1.2	Vu03
VrAUX-IAA-13	Vigun03g022700.1.v1.2	Vu03
VrARF-19	Vigun03g172200.1.v1.2	Vu03
VrARF-21	Vigun03g052100.2.v1.2	Vu03
VrAUX-IAA-19	Vigun03g226300.1.v1.2	Vu03
VrARF-4	Vigun04g001300.1.v1.2	Vu04
Vr-AUX-IAA-1	Vigun04g011800.1.v1.2	Vu04
VrAUX-IAA-8	Vigun04g011800.1.v1.2	Vu04
VrAUX-IAA-13	Vigun04g011800.1.v1.2	Vu04
VrAUX-IAA-17	Vigun04g011800.1.v1.2	Vu04
Vr-AUX-IAA-1	Vigun05g283400.1.v1.2	Vu05
VrARF-9	Vigun05g247900.1.v1.2	Vu05
VrARF-8	Vigun05g250500.1.v1.2	Vu05
VrARF-13	Vigun05g247900.1.v1.2	Vu05
VrARF-12	Vigun05g250500.1.v1.2	Vu05
VrAUX-IAA-9	Vigun05g280100.1.v1.2	Vu05
VrAUX-IAA-8	Vigun05g283400.1.v1.2	Vu05
VrAUX-IAA-7	Vigun05g288100.1.v1.2	Vu05
VrAUX-IAA-13	Vigun05g283400.1.v1.2	Vu05
VrARF-22	Vigun05g247900.1.v1.2	Vu05
VrAUX-IAA-18	Vigun05g280100.1.v1.2	Vu05
VrAUX-IAA-17	Vigun05g283400.1.v1.2	Vu05
VrARF-17	Vigun06g093900.1.v1.2	Vu06

VrAUX-IAA-15	Vigun06g113100.1.v1.2	Vu06
VrAUX-IAA-14	Vigun06g113100.1.v1.2	Vu06
VrARF-24	Vigun06g181000.1.v1.2	Vu06
VrARF-25	Vigun06g196900.2.v1.2	Vu06
VrARF-26	Vigun06g239000.1.v1.2	Vu06
VrARF-23	Vigun06g093900.1.v1.2	Vu06
VrARF-9	Vigun07g070700.1.v1.2	Vu07
VrAUX-IAA-4	Vigun07g131700.1.v1.2	Vu07
VrAUX-IAA-3	Vigun07g104300.1.v1.2	Vu07
VrAUX-IAA-3	Vigun07g155400.2.v1.2	Vu07
VrARF-13	Vigun07g070700.1.v1.2	Vu07
VrARF-13	Vigun07g201600.1.v1.2	Vu07
VrAUX-IAA-12	Vigun07g131700.1.v1.2	Vu07
VrAUX-IAA-15	Vigun07g155400.2.v1.2	Vu07
VrAUX-IAA-16	Vigun07g170800.1.v1.2	Vu07
VrARF-22	Vigun07g201600.1.v1.2	Vu07
VrAUX-IAA-14	Vigun07g104300.1.v1.2	Vu07
VrARF-15	Vigun08g094000.1.v1.2	Vu08
VrARF-18	Vigun08g008700.1.v1.2	Vu08
VrARF-17	Vigun08g139100.1.v1.2	Vu08
VrARF-16	Vigun08g188400.1.v1.2	Vu08
VrARF-23	Vigun08g139100.1.v1.2	Vu08
VrAUX-IAA-9	Vigun10g179000.1.v1.2	Vu10
VrAUX-IAA-8	Vigun10g181600.3.v1.2	Vu10
VrAUX-IAA-7	Vigun10g186100.1.v1.2	Vu10
VrAUX-IAA-18	Vigun10g179000.1.v1.2	Vu10
VrAUX-IAA-17	Vigun10g181600.3.v1.2	Vu10
VrARF-8	Vigun11g146300.1.v1.2	Vu11
VrARF-9	Vigun11g141500.1.v1.2	Vu11
VrARF-12	Vigun11g146300.1.v1.2	Vu11
VrARF-13	Vigun11g141500.1.v1.2	Vu11
