# Supplementary material for: Analysis of Auxin-Encoding Gene Family in Vigna radiata and It’s Cross-Species Expression Modulating Waterlogging Tolerance in Wild Vigna umbellata
Source: Plants (Basel). 2023 Nov 15;12(22):3858. doi: 10.3390/plants12223858 (PMC10674698; doi:10.3390/plants12223858)
Supplement: Supplementary file 1 [file plants-12-03858-s001.zip › Table S4.pdf]

Table S4: Synteny analysis of VrAUX-IAA and VrARF genes with *Medicago truncatula*

**Auxin responsive genes in *Vigna radiata***	**Gene ID (Orthologous) from *Medicago truncatula***	**Chromosome number**
VrARF-9	Medtr7g101280.1.JCVIMt4.0v1	chr7
VrAUX-IAA-4	Medtr7g110790.1.JCVIMt4.0v1	chr7
VrAUX-IAA-3	Medtr7g096090.1.JCVIMt4.0v1	chr7
VrAUX-IAA-12	Medtr7g110790.1.JCVIMt4.0v1	chr7
VrARF-4	Medtr8g101360.1.JCVIMt4.0v1	chr8
Vr-AUX-IAA-1	Medtr8g103030.1.JCVIMt4.0v1	chr8
VrAUX-IAA-9	Medtr8g014520.1.JCVIMt4.0v1	chr8
VrARF-21	Medtr8g100050.1.JCVIMt4.0v1	chr8
VrAUX-IAA-13	Medtr8g103030.1.JCVIMt4.0v1	chr8
VrAUX-IAA-18	Medtr8g014520.1.JCVIMt4.0v1	chr8
VrAUX-IAA-17	Medtr8g103030.1.JCVIMt4.0v1	chr8
VrAUX-IAA-19	Medtr8g067530.1.JCVIMt4.0v1	chr8
VrAUX-IAA-6	Medtr5g067350.1.JCVIMt4.0v1	chr5
VrARF-17	Medtr5g076270.1.JCVIMt4.0v1	chr5
VrARF-23	Medtr5g076270.1.JCVIMt4.0v1	chr5
VrAUX-IAA-19	Medtr5g030710.1.JCVIMt4.0v1	chr5
Vr-AUX-IAA-1	Medtr6g488150.1.JCVIMt4.0v1	chr6
VrAUX-IAA-8	Medtr6g488150.1.JCVIMt4.0v1	chr6
VrAUX-IAA-13	Medtr6g488150.1.JCVIMt4.0v1	chr6
VrAUX-IAA-17	Medtr6g488150.1.JCVIMt4.0v1	chr6
VrARF-15	Medtr3g073420.1.JCVIMt4.0v1	chr3
VrARF-17	Medtr3g064050.1.JCVIMt4.0v1	chr3
VrAUX-IAA-10	Medtr3g106850.1.JCVIMt4.0v1	chr3
VrARF-23	Medtr3g064050.1.JCVIMt4.0v1	chr3
VrARF-9	Medtr4g058930.1.JCVIMt4.0v1	chr4
VrARF-8	Medtr4g060460.1.JCVIMt4.0v1	chr4
VrARF-13	Medtr4g058930.1.JCVIMt4.0v1	chr4
VrARF-12	Medtr4g060460.1.JCVIMt4.0v1	chr4
VrARF-14	Medtr4g124900.1.JCVIMt4.0v1	chr4
VrAUX-IAA-7	Medtr4g011880.1.JCVIMt4.0v1	chr4
VrAUX-IAA-10	Medtr4g115075.1.JCVIMt4.0v1	chr4
VrAUX-IAA-12	Medtr4g124300.1.JCVIMt4.0v1	chr4
VrARF-20	Medtr4g124900.1.JCVIMt4.0v1	chr4
VrAUX-IAA-11	Medtr4g128070.1.JCVIMt4.0v1	chr4
VrARF-25	Medtr4g088210.1.JCVIMt4.0v1	chr4
VrAUX-IAA-4	Medtr1g085750.1.JCVIMt4.0v1	chr1
VrAUX-IAA-3	Medtr1g093240.2.JCVIMt4.0v1	chr1
VrARF-13	Medtr1g064430.2.JCVIMt4.0v1	chr1
VrARF-18	Medtr1g024025.1.JCVIMt4.0v1	chr1
VrAUX-IAA-12	Medtr1g085750.1.JCVIMt4.0v1	chr1
VrARF-22	Medtr1g094960.1.JCVIMt4.0v1	chr1

VrARF-22	Medtr1g064430.2.JCVIMt4.0v1	chr1
VrAUX-IAA-16	Medtr1g093240.2.JCVIMt4.0v1	chr1
VrAUX-IAA-15	Medtr1g080860.1.JCVIMt4.0v1	chr1
VrAUX-IAA-14	Medtr1g070520.1.JCVIMt4.0v1	chr1
Vr-AUX-IAA-1	Medtr2g101500.1.JCVIMt4.0v1	chr2
VrARF-8	Medtr2g093740.2.JCVIMt4.0v1	chr2
VrARF-9	Medtr2g094570.1.JCVIMt4.0v1	chr2
VrARF-12	Medtr2g093740.2.JCVIMt4.0v1	chr2
VrARF-13	Medtr2g094570.1.JCVIMt4.0v1	chr2
VrAUX-IAA-9	Medtr2g100780.2.JCVIMt4.0v1	chr2
VrAUX-IAA-8	Medtr2g101500.1.JCVIMt4.0v1	chr2
VrARF-14	Medtr2g043250.1.JCVIMt4.0v1	chr2
VrARF-20	Medtr2g043250.1.JCVIMt4.0v1	chr2
VrAUX-IAA-13	Medtr2g101500.1.JCVIMt4.0v1	chr2
VrARF-22	Medtr2g094570.1.JCVIMt4.0v1	chr2
VrAUX-IAA-18	Medtr2g100780.2.JCVIMt4.0v1	chr2
VrAUX-IAA-17	Medtr2g101500.1.JCVIMt4.0v1	chr2
VrARF-26	Medtr2g005240.1.JCVIMt4.0v1	chr2
VrARF-25	Medtr2g014770.1.JCVIMt4.0v1	chr2
VrARF-24	Medtr2g018690.1.JCVIMt4.0v1	chr2
