# Supplementary material for: Analysis of Auxin-Encoding Gene Family in Vigna radiata and It’s Cross-Species Expression Modulating Waterlogging Tolerance in Wild Vigna umbellata
Source: Plants (Basel). 2023 Nov 15;12(22):3858. doi: 10.3390/plants12223858 (PMC10674698; doi:10.3390/plants12223858)
Supplement: Supplementary file 1 [file plants-12-03858-s001.zip › Table S5.pdf]

Table S5: Synteny analysis of VrAUX-IAA and VrARF genes with *Arabidopsis thaliana*

**Auxin responsive genes in *Vigna radiata***	**Gene ID (Orthologous) from *Arabidopsis thaliana***	**Chromosome number**
VrAUX-IAA-3	AT1G04250.1.TAIR10	Chr1
VrAUX-IAA-7	AT1G15580.1.TAIR10	Chr1
VrAUX-IAA-7	AT1G52830.1.TAIR10	Chr1
VrARF-15	AT1G77850.1.TAIR10	Chr1
VrARF-14	AT1G19220.1.TAIR10	Chr1
VrARF-18	AT1G19850.1.TAIR10	Chr1
VrARF-20	AT1G19220.1.TAIR10	Chr1
VrARF-9	AT2G28350.1.TAIR10	Chr2
VrAUX-IAA-4	AT2G46990.1.TAIR10	Chr2
VrARF-13	AT2G28350.1.TAIR10	Chr2
VrAUX-IAA-6	AT2G22670.4.TAIR10	Chr2
VrARF-25	AT2G33860.1.TAIR10	Chr2
VrAUX-IAA-19	AT2G22670.4.TAIR10	Chr2
VrAUX-IAA-3	AT3G23050.1.TAIR10	Chr3
VrAUX-IAA-4	AT3G62100.1.TAIR10	Chr3
VrAUX-IAA-7	AT3G15540.1.TAIR10	Chr3
VrAUX-IAA-12	AT3G17600.1.TAIR10	Chr3
VrAUX-IAA-16	AT3G23050.1.TAIR10	Chr3
VrAUX-IAA-15	AT3G04730.1.TAIR10	Chr3
VrAUX-IAA-14	AT3G23050.1.TAIR10	Chr3
VrAUX-IAA-3	AT4G14550.1.TAIR10	Chr4
VrAUX-IAA-8	AT4G29080.1.TAIR10	Chr4
VrAUX-IAA-10	AT4G32280.1.TAIR10	Chr4
VrAUX-IAA-14	AT4G14550.1.TAIR10	Chr4
VrAUX-IAA-16	AT4G14550.1.TAIR10	Chr4
VrARF-8	AT5G60450.1.TAIR10	Chr5
VrARF-12	AT5G60450.1.TAIR10	Chr5
VrAUX-IAA-6	AT5G65670.1.TAIR10	Chr5
VrARF-14	AT5G20730.1.TAIR10	Chr5
VrARF-17	AT5G37020.1.TAIR10	Chr5
VrARF-20	AT5G20730.1.TAIR10	Chr5
VrAUX-IAA-11	AT5G57420.1.TAIR10	Chr5
VrAUX-IAA-18	AT5G25890.1.TAIR10	Chr5
VrAUX-IAA-19	AT5G65670.1.TAIR10	Chr5
