# Supplementary material for: Analysis of Auxin-Encoding Gene Family in Vigna radiata and It’s Cross-Species Expression Modulating Waterlogging Tolerance in Wild Vigna umbellata
Source: Plants (Basel). 2023 Nov 15;12(22):3858. doi: 10.3390/plants12223858 (PMC10674698; doi:10.3390/plants12223858)
Supplement: Supplementary file 1 [file plants-12-03858-s001.zip › Table S6.pdf]

Table S6: Primers used in the expression profiling

Gene code Name	Sequence (5′-3′) of forward and reverse primers
VrARF-25	GCAGCCCAGTTGAAAGGTTCTG
	CGC TCT GCA GCA TCT TCT GT
VrARF-5	CGC TTC TCC GTC ACA TTC TC
	AAC TCG CGC TCT TCG TCG TC
VrARF-22	GGC CAA ACC AGG GTT CCA CC
	GGA GGC TCA GCT GAA TAG TC
VrARF-4	GGA GCT GTG GCA AGC TTG TG
	CTC CGT GAG TGC TGG TAT CA
VrARF-11	GCA GAA CTA CAA CCT ACT CC
	AAG TGT CGT TTC GGC TGA C
VrAUX-IAA-13	AGG GCG TGA GTC ACC TGA G
	GCC TTG GCA GCT GGA GCA GA
VrAUX-IAA-7	GCA GAT CAG ACG GAG AGG AT
	GGT AGG AGC AAA CCG GAG GC
VrAUX-IAA-9	GAC TGG TCT CTC GGT GGT AA
	GCT ATC ACC ACC ACC ACA AC
VrAUX-IAA-2	TAC AGG AAC GTC CTG CTG CA
	CAT CTC CAA CAA GCA TCC AG
VrAUX-IAA-15	CAC TGG TGG CAA GAG AGG
	CCA TGT CTC AGA CAA GAT CC
